# Effects of intervention combining transcranial direct current stimulation and foot core exercise on sensorimotor function in foot and static balance

**DOI:** 10.1186/s12984-022-01077-5

**Published:** 2022-09-14

**Authors:** Songlin Xiao, Baofeng Wang, Changxiao Yu, Bin Shen, Xini Zhang, Dongqiang Ye, Liqin Deng, Yongxin Xu, Junhong Zhou, Weijie Fu

**Affiliations:** 1 Key Laboratory of Exercise and Health Sciences of Ministry of Education, Shanghai University of Sport, Shanghai, 200438 China; 2 The Hinda and Arthur Marcus Institute for Aging Research, Hebrew SeniorLife, Boston, MA 02131 USA; 3 Harvard Medical School, Boston, MA 02131 USA; 4 Shanghai Frontiers Science Research Base of Exercise and Metabolic Health, Shanghai University of Sport, Shanghai, 200438 China

**Keywords:** Transcranial direct current stimulation, Foot core exercise, Foot muscle strength, Passive ankle kinesthesia, Static balance

## Abstract

**Objective:**

This study aimed to examine the effects of combining transcranial direct current stimulation (tDCS) and foot core exercise (FCE) on the sensorimotor function of the foot (i.e., toe flexor strength and passive ankle kinesthesia) and static balance.

**Methods:**

In this double-blinded and randomized study, 30 participants were randomly assigned into two groups: tDCS combined with FCE and sham combined with FCE (i.e., control group). The participants received 2 mA stimulation for 20 min concurrently with FCE over 4 weeks (i.e., three sessions per week). After the first two groups completed the intervention, a reference group (FCE-only group) was included to further explore the placebo effects of sham by comparing it with the control group. Foot muscle strength, passive ankle kinesthesia, and static balance were assessed at baseline and after the intervention.

**Results:**

Compared with the control group and baseline, tDCS combined with FCE could increase toe flexor strength (*p* < 0.001) and decrease the passive kinesthesia threshold of ankle eversion (*p* = 0.002). No significant differences in static balance were observed between tDCS + FCE and control groups. The linear regression models showed an association towards significance between the percent changes in metatarsophalangeal joint flexor strength and the anteroposterior average sway velocity of the center of gravity in one-leg standing with eyes closed following tDCS + FCE (*r*^*2*^ = 0.286; *p* = 0.057). The exploratory analysis also showed that compared with FCE alone, the sham stimulation did not induce any placebo effects during FCE.

**Conclusion:**

Participating in 4 weeks of intervention using tDCS in combination with FCE effectively enhances toe flexor strength and foot–ankle sensory function.

## Introduction

The feet are the only part of the body in direct contact with the ground when standing and walking, helping support body weight and controlling posture [1]. Maintaining the intact sensorimotor function of the feet is critical to safe standing and walking. The diminished muscle strength and somatosensory function of the foot and ankle have been linked to poor functional performance and increased risk of injuries (e.g., chronic ankle instability) and falls [2, 3]. Therefore, augmenting the sensorimotor function of the foot is highly demanded, which could ultimately benefit physical function and help prevent relevant risk events.

The regulation of foot function depends upon peripheral and central elements. Musculoskeletal strength is one of the peripheral elements contributing to foot function. Interventions to augment foot muscle strength could enhance foot function and thus help prevent injuries to the foot and ankle [2, 4]. Previous studies showed that daily foot core exercise (FCE) could improve the intrinsic foot muscle strength and thus help enhance jumping and postural control performance [5–7]. Meanwhile, in regulating foot function, the mechanoreceptors within the soles of the foot perceive the sensory information from the environment, and such afferent information is delivered to the supraspinal regions via peripheral nerves and spinal cord and activates a distributed cortical network within the brain [8]. The somatosensory information is integrated with other sensory inputs within the cortical networks and forms volitional movements in daily activities [9, 10]. Researchers have linked the activation of sensorimotor cortical regions (i.e., central elements) to the sensorimotor function in the lower extremities [8, 11]. The decrease in the excitability of sensorimotor cortical regions, as induced by chronic ankle instability (CAI), contributes to persistent aberrant biomechanical patterns and predisposing individuals to further bouts of instability and re-injury [12]. Therefore, strategies designed to facilitate the sensorimotor cortical network of the brain are also of great promise to restore/augment the sensorimotor function and benefit the functional performance.

Transcranial direct current stimulation (tDCS) could invasively modulate the cortical excitability by sending low-amplitude direct currents to targeting cortical regions between two or more electrodes placed on the scalp [13]. Several piloting studies have shown the effects of tDCS targeting sensorimotor regions on the lower-extremity function, balance, and mobility [8, 11, 14]. One study showed that one session of 20-min tDCS applied over the sensorimotor cortex could enhance toe pinch force in healthy young adults [11]. Other studies demonstrated that using tDCS could decrease the vibrotactile threshold of the foot sole when standing and the tactile threshold of the left center of the distal pulp of the hallux [8, 15]. These findings suggested that tDCS could potentially enhance the muscle strength and somatosensory function of the foot by targeting the central elements.

However, in previous studies, only the effects of intervention targeting either peripheral or central elements on the sensorimotor function of the foot were examined. Interventions simultaneously targeting peripheral and central elements could induce greater benefits than traditional “single-target” interventions [16]. Therefore, in the present study, a novel intervention targeting the peripheral and central elements of sensorimotor regulation was designed by combining FCE and tDCS (i.e., combined intervention), and this combined intervention could induce greater benefits on the sensorimotor function of the foot and the related functional performance (e.g., static balance) than FCE.

In this randomized, double-blinded, and sham-controlled study, we examined the effects of combining sensorimotor tDCS and FCE on the foot sensorimotor function (i.e., toe flexor strength and passive ankle kinesthesia) and static balance and further explored the associations between changes in foot sensorimotor function and changes in static balance. The hypotheses are as follows: (1) compared with the control (i.e., sham stimulation with FCE), the combined intervention could induce significant improvements in the sensorimotor function of the foot and static balance in healthy younger adults, and (2) such augmentation of the sensorimotor function of the foot induced by the combined intervention could be associated with the extent in the improvements of standing static balance. Secondarily, a reference group who completed FCE only was included to examine if sham stimulation induced placebo effects.

## Methods

### Participants

Previous studies using in the combination of tDCS with exercise with similar outcome variables (i.e., balance and proprioception) were used to estimate a sample size. The values of the effect size (${\eta}_{p}^{2}$ = 0.096–0.129) were observed in these studies, which is equal to the effect size values of f (f = 0.33–0.38) [17, 18]. Thus, the sample size was calculated using a power analysis with a statistical power of 0.80, a probability level of 0.05, and an effect size of 0.33 via G*Power 3.1.9.2 software. Eleven participants per group were identified as achieving sufficient power. Thus, a total of 30 participants was recruited, randomly divided into two groups, to account for up to 25 percent attrition and partly reduce the risk of underestimating the sample size. Healthy younger adults without the habit of regular exercise were recruited from a university community via advertisements. The inclusion criteria were as follows: ability to stand or walk without any personal assistance and no history of lower extremity injuries in the past 6 months. The exclusion criteria were as follows: skin allergies, using neuropsychiatric medication, overt neurological disease, any contraindications to the use of tDCS (e.g., metal-implanted devices in the brain), and participation in another kind of training program on foot sensorimotor function. All participants provided a written informed consent as approved by the Institutional Review Board of the Shanghai University of Sport (No.102772021RT035) to participate in this study.

### Study design

**Fig. 1:**
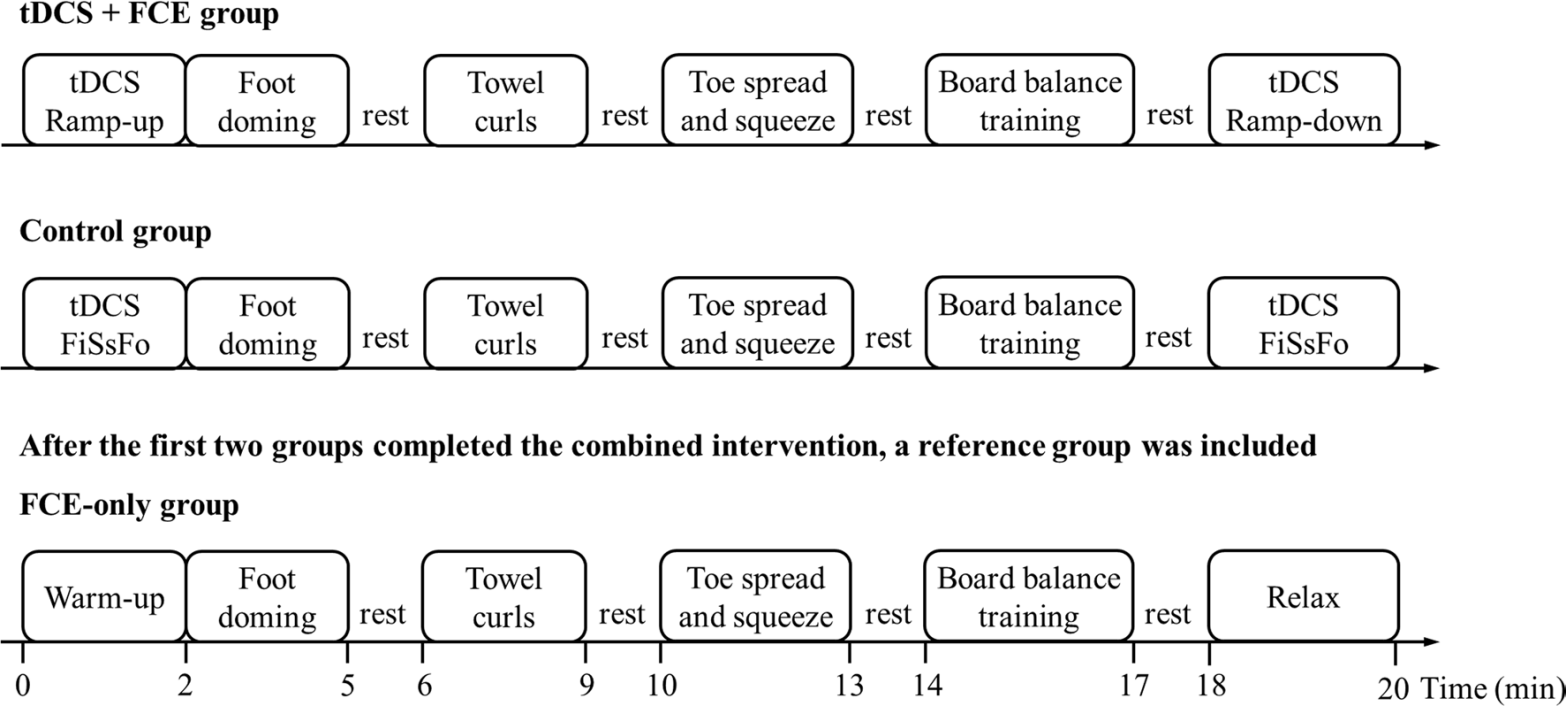
Intervention protocols. *FCE* foot core exercise, *FiSsFo* fade-in short stimulation fade-out approach

After screening was performed, 30 participants were randomly assigned to two groups (*n* = 15 in each group, Table 1) using a Microsoft Excel random number table: tDCS combined with FCE (tDCS + FCE group), and sham combined with FCE (control group). The participants in the tDCS + FCE group performed FCE while receiving tDCS, while those in the control group performed FCE while receiving sham stimulation (Fig. 1). A total of 12 training sessions were performed over 4 weeks (i.e., three sessions per week). Within each week, at least 1 day of resting was provided between sessions. Foot muscle strength, passive ankle kinesthesia, and static balance were assessed at baseline and within 24 h after the last session of the 4-week intervention.

**Table 1:** Basic information of participants

Groups	Year (yrs)	Height (cm)	Weight (kg)
tDCS + FCE group (*n* = 15)	20.5 ± 1.8	177.5 ± 8.1	71.7 ± 10.0
Control group (*n* = 15)	21.3 ± 1.8	176.9 ± 6.4	73.1 ± 10.6
*p*	0.235	0.804	0.721

Then, to test the secondary exploratory hypothesis on placebo effects, 15 participants were additionally recruited as the reference group (age: 22.3 ± 2.3 years; height: 174.7 ± 8.8 cm; weight: 69.0 ± 12.6 kg) following the same inclusion/exclusion criteria. The participants in the reference group completed FCE only, with the same training schedule as the control group (Fig. 1). For successful blinding, this intervention was completed after the tDCS + FCE and control groups completed the study, and the participants in the reference group did not know the study protocol in those two groups.

### High-definition transcranial direct current stimulation

**Fig. 2:**
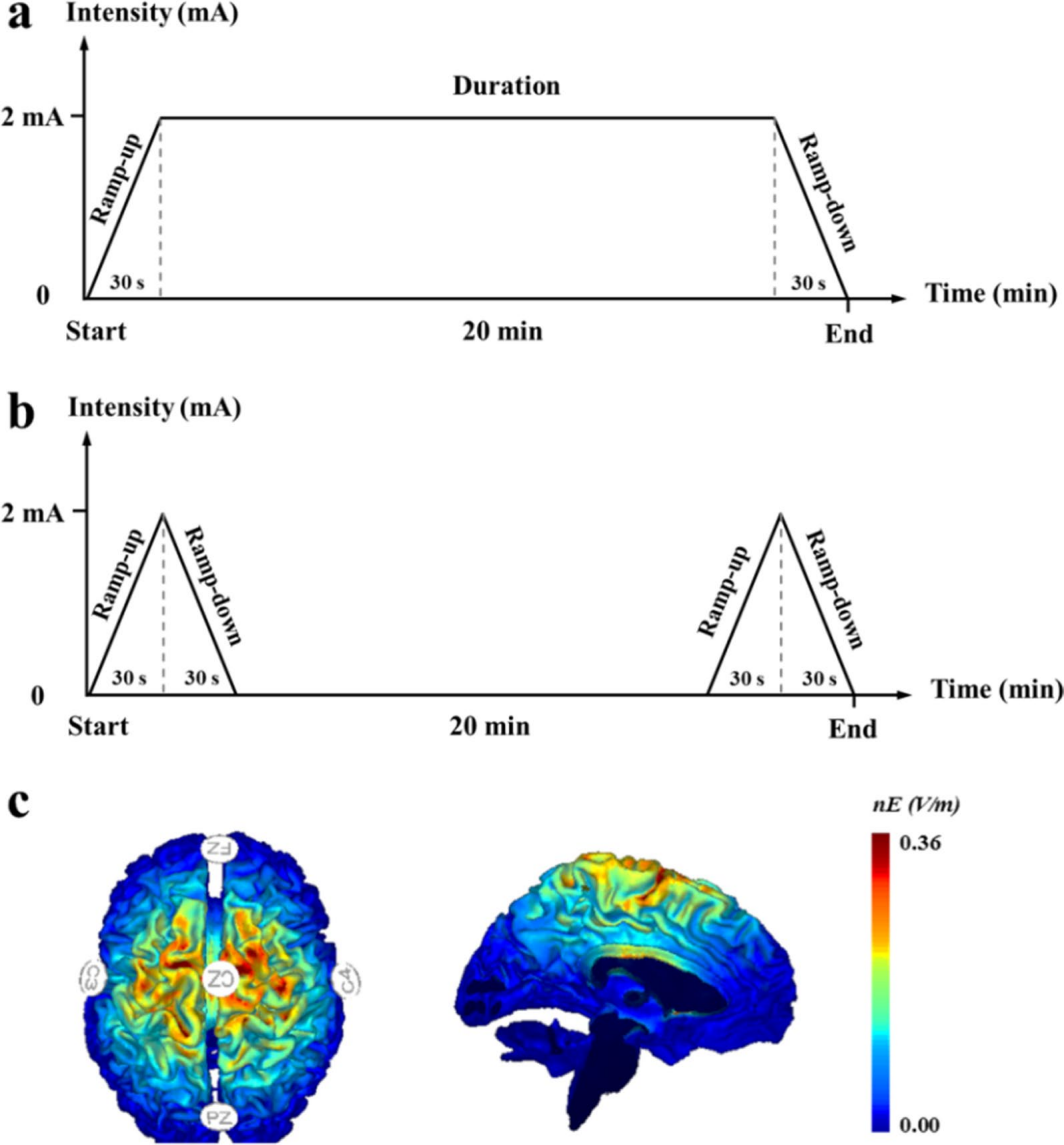
High-definition transcranial direct current stimulation. **a** tDCS protocol, **b** sham protocol, and **c** electrode montages for tDCS and simulated distribution of electrical field in the brain

tDCS was administered with a battery-driven, wireless multichannel Starstim® neurostimulator system (Neuroelectrics, Barcelona, Spain). A 4 × 1 ring-type high-definition tDCS (HD-tDCS) montage was used [19]. The current was delivered using round gel Ag/AgCl electrodes (3.14 cm^2^). The anodal electrode was placed over the Cz and was surrounded by four return electrodes (i.e., C3, C4, Fz, and Pz) on the basis of 10/20 electroencephalogram (EEG) brain templates. The maximum current intensity of this HD-tDCS was set at 2 mA and the stimulation duration was 20 min. The current intensity was ramped up from 0 to 2 mA in the initial 30 s at the beginning of the stimulation and ramped down to 0 in the last 30 s of the stimulation [20] (Fig. 2a). The montage (e.g., electrode placement) of sham was the same as that of tDCS, but the current was delivered within the first and last 60 s of stimulation (i.e., 30-s ramp up and 30-s ramp down, Fig. 2b) [20]. This HD-tDCS montage activates the sensorimotor cortical regions of the lower-extremity area (Fig. 2c). The stimulation condition (i.e., tDCS and sham) was coded at the beginning of the study and only known by a person who was not involved in any of the study procedures. Then, the condition was programmed in the system before the stimulation session in a blinded mode so that neither participant nor study personnel knew the stimulation that was applied. The participants were asked to complete a questionnaire at the end of each stimulation visit to evaluate the potential side effects. They were also asked to assume if they received tDCS or sham after the last session of intervention to assess the blinding efficacy.

### Foot core exercise

The FCE in this study consisted of foot doming, towel curls, toe spread and squeeze, and balance board training, with a goal to strengthen intrinsic and extrinsic foot muscles and the functionalities of the foot and ankle [7]. All participants were verbally instructed, provided with a demonstration, and guided through a single practice trial. Following the instruction, the participants sequentially performed each exercise to the best of their ability in barefoot (Table 2).

**Table 2:** Daily foot core exercise progression

Training	Week 1	Week 2	Week 3	Week 4
Foot doming	2 sets of 10 times	2 sets of 15 times	2 sets of 20 times	3 sets of 20 times
Towel curls	3 sets of 10 times	3 sets of 20 times	3 sets of 10 times (0.25 kg)	3 sets of 15 times (0.5 kg)
Toe spread and squeeze	2 sets of 10 times	2 sets of 15 times	2 sets of 20 times	3 sets of 20 times
Balance board training	2 sets of 20 s	2 sets of 25 s	2 sets of 30 s	3 sets of 30 s

### Data collection

#### Passive ankle kinesthesia

The passive kinesthesia threshold of the ankle joint was assessed using an ankle proprioception tester (KP-11, Toshimi, Shandong, China) [21]. In particular, each participant sat on an adjustable seat, and the dominant foot was placed on the bottom of the foot pedal. Only half of the weight in the lower extremity was loaded onto the platform. Each participant wore an eye mask and noise reduction earphones during the test. The platform was randomly activated to drive the participant’s ankle in plantarflexion (PF), dorsiflexion (DF), inversion (INV), and eversion (EV). After the trigger and the direction of foot movement were confirmed, the participants pressed the stop button immediately. The research personnel then recorded the angular displacement. The participants completed three trials of the test in each movement direction (i.e., PF, DF, INV, and EV) in a randomized order. A rest period of 1 min was provided between trials.

### Metatarsophalangeal joint (MPJ) flexor strength

MPJ flexor strength was measured using an MPJ flexor strength testing system customized and validated by the team [22, 23]. Each participant was seated in the system with bare feet. The position and height of the seat were adjusted to make the thighs parallel to the ground, and the knee joint was fixed at 90°. The participants were asked to flex the MPJ and press the pedal for 10 s with maximum force. The measurement was repeated thrice, with a rest period of 1 min. The peak MPJ flexor strength was then obtained and normalized in accordance with the body weight of each participant.

### Toe flexor strength

Toe flexor strength was measured in sitting position by using a toe grip dynamometer (T.K.K.3361, Takei Scientific Instruments Co., Niigata, Japan) [24, 25]. Each participant was asked to sit on an adjustable seat, and the dominant foot was placed on the dynamometer and fixed with the heel stopper. The participants were asked to flex their toes vigorously for at least 3 s. The toe flexor strength was recorded and normalized by the body weight of each participant. The measurement was repeated thrice, with an interval of 1 min.

### Static balance

In the standing balance test, each participant stood barefoot on the balance testing system (Super Balance, Acmeway, Beijing, China), and the feet were apart shoulder width. The participants were asked to keep their eyes on a horizontal level. They completed three trials within each of the following conditions: two-leg standing with eyes open (TL_EO) and eyes closed (TL_EC) and one-leg standing with eyes open (OL_EO) and eyes closed (OL_EC). Each of the two-leg standing trials lasted 30 s, and the one-leg trial lasted 10 s. A break of 30 s was provided between trials. The system recorded the sway velocity of the center of gravity (CoG) in the medial–lateral (ML) and anteroposterior (AP) directions.

### Statistics

SPSS 22.0 (SPSS Inc., Chicago, IL, USA) was used to complete the statistical analysis, and all data were expressed by mean ± standard deviation. Shapiro–Wilk test was used to examine if the outcomes were normally distributed. Data with non-normal distribution were converted into normal distribution by the logarithmic transformation [26]. Independent-sample *t*-test was used to assess the significant difference in the outcomes at baseline between the tDCS and control groups and between the control and reference groups.

Two-way repeated measures ANOVA models were used to examine the effects of the combined intervention on the sensorimotor function of the foot and static balance. The model factors were condition (i.e., tDCS and sham), time (baseline and follow-up), and their interaction. The dependent variable was each functional outcome in separate models. Similar ANOVA models were used to explore the potential placebo effects of sham stimulation. The model factors were group (i.e., sham + FCE and FCE-only), time (baseline and follow-up), and their interaction. Bonferroni’s multiple comparisons tests were used as post-hoc analyses to determine the location where the significance was in the ANOVA models. The effect size value (${\eta}_{\text{p}}^{2}$) was reported to ANOVAs and the effect size value (Cohen’s *d*) was reported to the post-hoc tests.

Linear regression analyses were conducted to examine the relationship between the percent changes in the sensorimotor function of the foot induced by combined intervention and the percent changes in static balance. Extreme outliers were removed using the Tukey outlier method of 1.5 × IQR. The significance level was set to *p* < 0.05 for all the analyses.

## Results

All the participants in the tDCS + FCE and control groups completed this study, and their data were collected successfully. One participant in the reference group dropped out due to a loss of interest. No side effects nor adverse events were reported. No significant differences in demographics and the functional performance at baseline were observed (*p* = 0.124–0.860). For blinding efficacy, 83.3% of the participants guessed that the intervention was tDCS, and only 16.7% guessed that it was sham (46.7% total error rate of guessing the type of tDCS). The passive kinesthesia thresholds of PF, DF, and INV, the AP and ML average CoG sway velocities in TL_EO, the AP average CoG sway velocities in OL_EO and OL_EC were not normally distributed. Thus, a logarithmic transformation was performed. By using the Tukey outlier method, two outliers were removed from the linear regression analyses.

### Effects of combined intervention on sensorimotor function of the foot and static balance

**Fig. 3:**
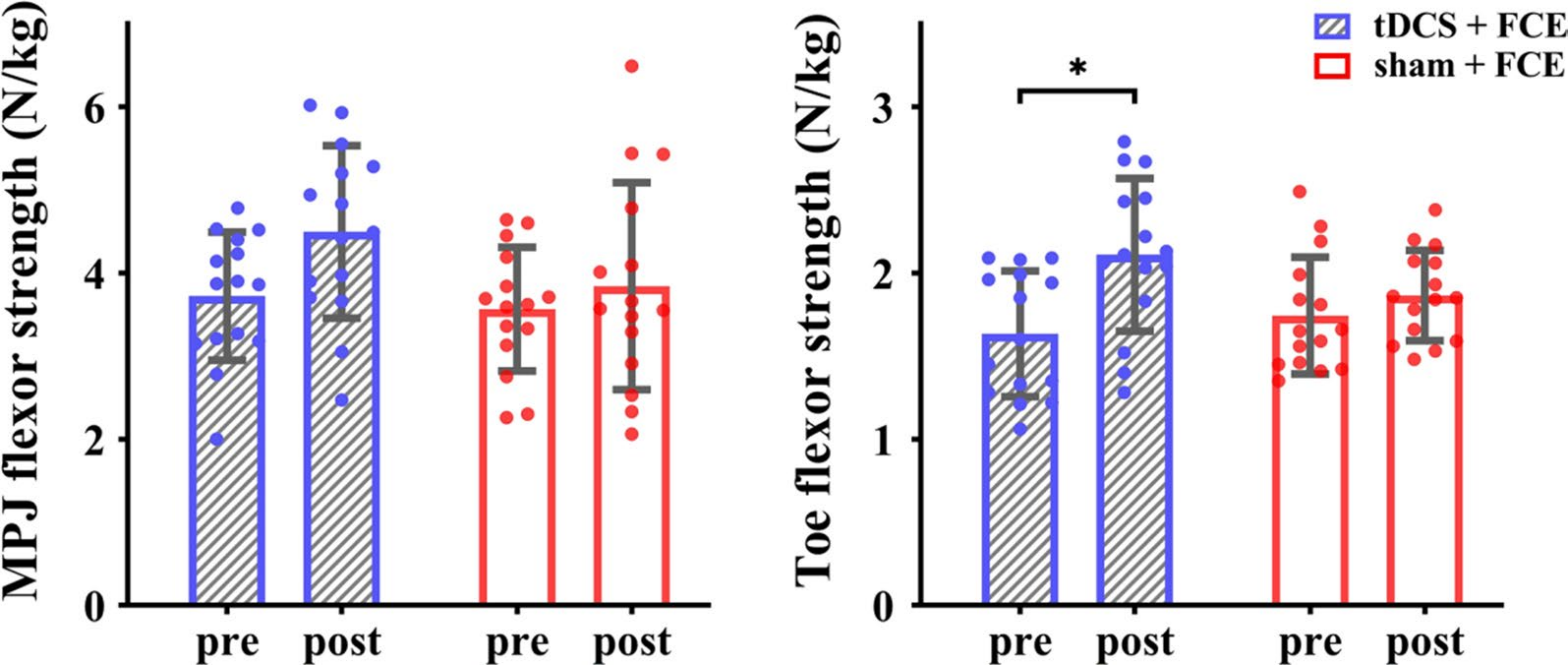
Effects of tDCS combined with FCE on foot muscle strength. *MPJ* metatarsophalangeal joint, *FCE* foot core exercise; *p < 0.05

The ANOVA models showed a significant interaction on toe flexor strength (*F*_(1, 28)_ = 12.359, *p* = 0.002, ${\eta}_{\text{p}}^{2}$ = 0.306). Post-hoc analysis showed that compared with the control group and baseline, tDCS + FCE induced a significant improvement in the toe flexor strength (t = 6.660, *p* < 0.001, Cohen’s *d* = 1.13, Fig. 3). In particular, the average percent changes in the toe flexor strength were 31% ± 19% in the tDCS + FCE group and only 9% ± 17% in the control group. Significant main effects of time were also observed for MPJ flexor strength (*F*_(1, 28)_ = 13.715, *p* = 0.001, ${\eta}_{\text{p}}^{2}$ = 0.329) and toe flexor strength (*F*_(1, 28)_ = 34.847, *p* < 0.001, ${\eta}_{\text{p}}^{2}$ = 0.554), indicating a significant increase from baseline to follow-up regardless of the group.

**Fig. 4:**
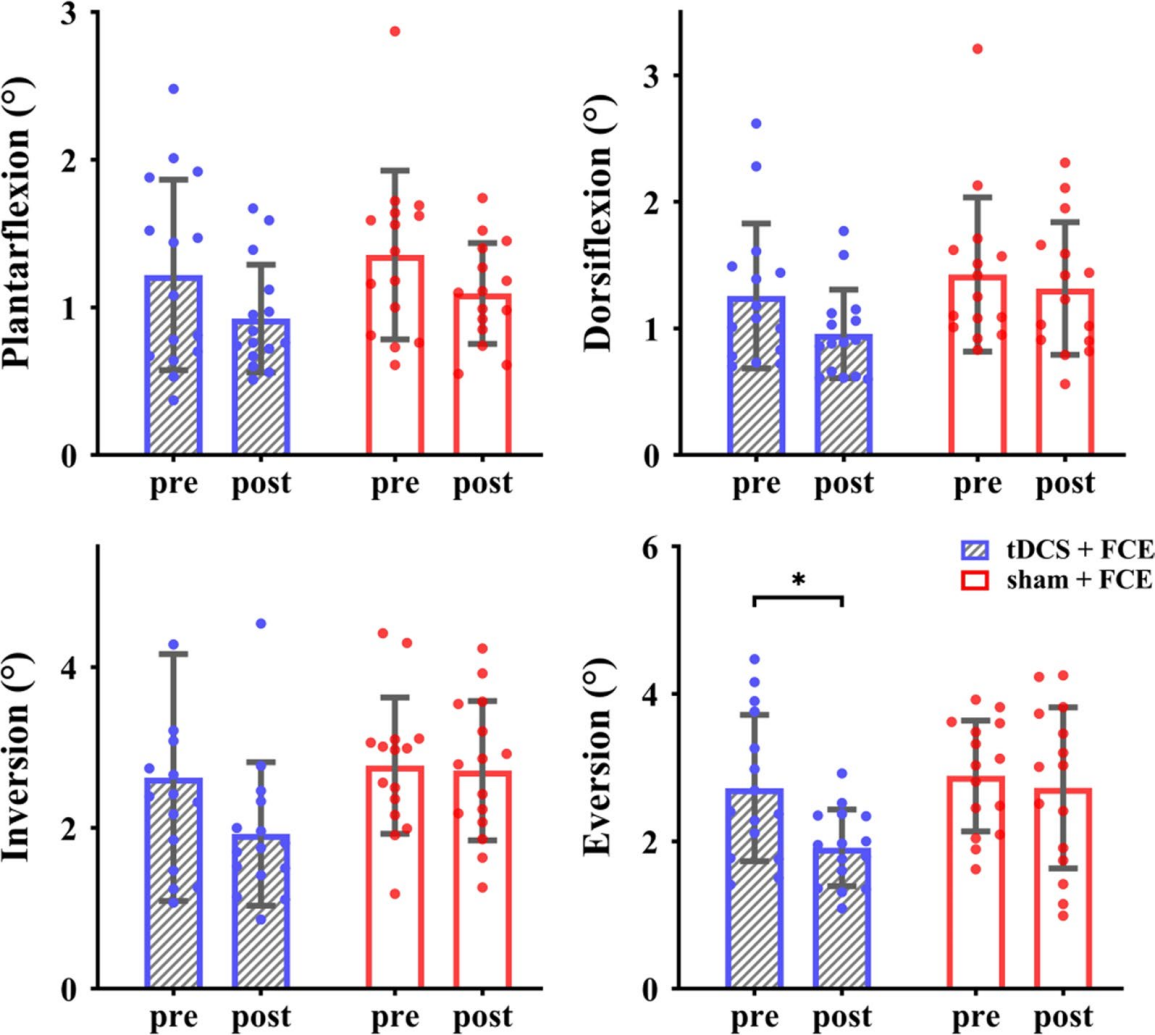
Effects of tDCS combined with FCE on passive ankle kinesthesia. *FCE* foot core exercise; *p < 0.05

A significant interaction on the passive kinesthesia threshold of EV (*F*_(1, 28)_ = 4.284, *p* = 0.048, ${\eta}_{\text{p}}^{2}$ = 0.133) was also observed. Post-hoc analysis showed that compared with the control group and baseline, the tDCS + FCE group demonstrated a significant decrease in the passive kinesthesia threshold of ankle eversion (t = 3.660, *p* = 0.002, Cohen’s *d* = 1.02, Fig. 4). In particular, the average percent changes in the passive kinesthesia threshold of ankle eversion were 25% ± 21% in the tDCS + FCE group and only 6% ± 32% in the control group. Significant main effects of time were also observed for the passive kinesthesia thresholds of PF (*F*_(1, 28)_ = 14.256, *p* = 0.001, ${\eta}_{\text{p}}^{2}$ = 0.337) and DF (*F*_(1, 28)_ = 7.445, *p* = 0.011, ${\eta}_{\text{p}}^{2}$ = 0.210), indicating a significant decrease from baseline to follow-up regardless of the group.

ANOVA revealed no significant intervention by time interaction effects for the variables of static balance ability (*p* > 0.05, Table 3). Only a significant main effect of time was observed for the AP average CoG sway velocity in OL_EC (*F*_(1, 28)_ = 5.097, *p* = 0.032, ${\eta }_{p}^{2}$ = 0.154), reflecting a significant decrease from baseline to follow-up regardless of the group.

**Table 3:** Effects of tDCS combined with FCE on static balance

Variables		tDCS + FCE group		Control group		*p* value		
Conditions	Sway velocity (mm/s)	Pre	Post	Pre	Post	Interaction effect	Main effect for time	Main effect for group
TL_EO	ML	8.37 ± 1.25	8.54 ± 0.85	8.92 ± 2.11	8.73 ± 1.28	0.764	0.515	0.620
	AP	9.57 ± 1.61	9.83 ± 1.26	10.22 ± 2.05	10.12 ± 1.32	0.595	0.680	0.359
TL_EC	ML	8.69 ± 1.50	9.00 ± 1.12	8.98 ± 1.88	8.89 ± 1.00	0.437	0.681	0.843
	AP	10.63 ± 1.94	10.73 ± 1.51	10.76 ± 2.02	10.52 ± 0.99	0.590	0.827	0.939
OL_EO	ML	19.91 ± 3.47	21.06 ± 5.05	18.65 ± 5.90	19.26 ± 4.12	0.948	0.237	0.235
	AP	31.99 ± 5.38	31.58 ± 6.37	29.62 ± 8.82	30.63 ± 6.49	0.471	0.762	0.481
OL_EC	ML	42.14 ± 12.99	37.39 ± 9.00	37.06 ± 17.25	35.58 ± 9.03	0.157	0.666	0.183
	AP	70.20 ± 16.83	60.73 ± 15.53	59.33 ± 22.47	56.31 ± 14.65	0.254	**0.032**	0.199

*FCE* foot core exercise, *TL_EO* two-leg standing with eyes open, *TL_EC* two-leg standing with eyes closed, *OL_EO* one-leg standing with eyes open, *OL_EC* one-leg standing with eyes closed, *ML* medial–lateral, *AP* anteroposterior, *CoG* the center of gravity

### Relationships between combined intervention-induced changes in the sensorimotor function of the foot and static balance

**Fig. 5:**
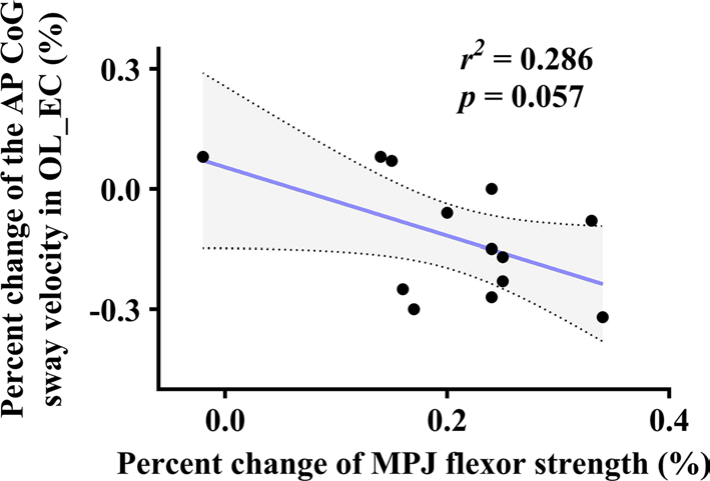
Relationship between combined intervention-induced changes in MPJ flexor strength and AP average CoG sway velocity in OL_EC. *MPJ* Metatarsophalangeal joint, *OL_EC* one-leg standing with eyes closed, *AP* anteroposterior, *CoG* the center of gravity

Within the tDCS + FCE group, the linear regression models showed an association towards significance between the percent changes in AP average CoG sway velocity in OL_EC and the changes in MPJ flexor strength (*r*^2^ =  0.286, *p* = 0.057, Fig. 5). Besides, within the control group, no significant association between the percent changes in AP average CoG sway velocity in OL_EC and the changes in MPJ flexor strength (*r*^*2*^ =  0.203, *p* = 0.122) was observed. Across both tDCS + FCE and control groups, no significant associations between intervention-induced changes in the sensorimotor function of the foot and static balance were observed (*p* > 0.05).

### Exploration of placebo effects

**Fig. 6:**
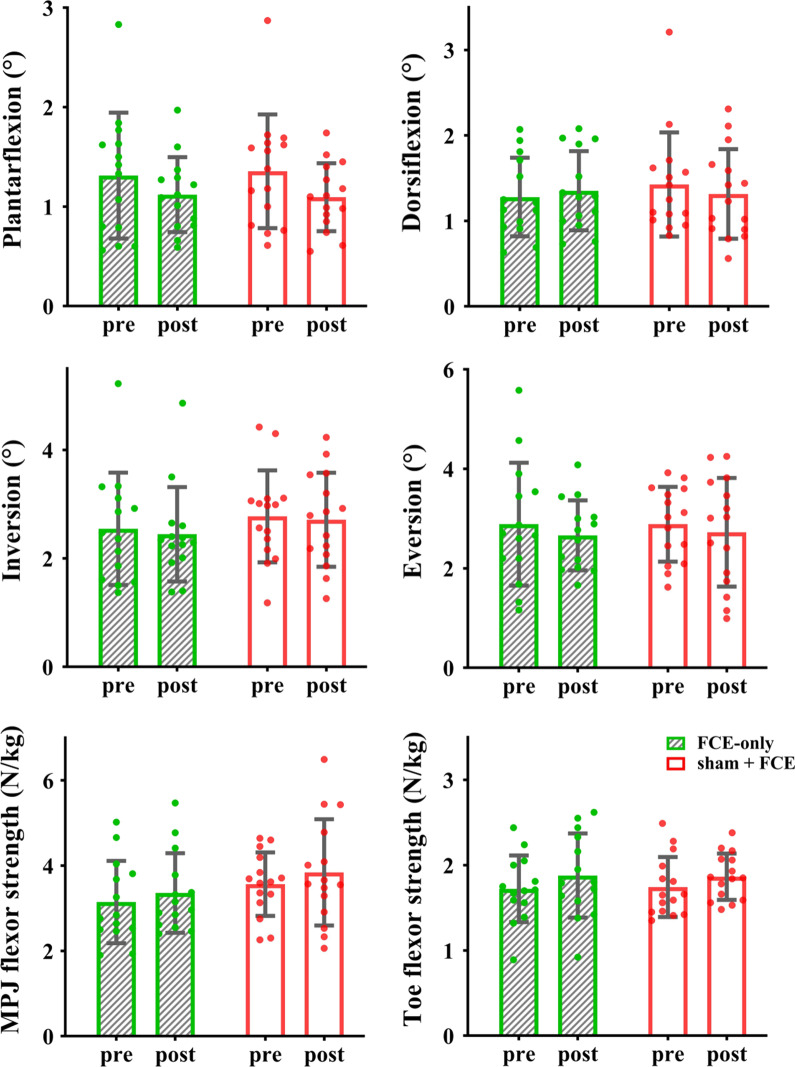
Comparisons of foot muscle strength and passive ankle kinesthesia thresholds between sham + FCE and FCE-only groups. *MPJ* metatarsophalangeal joint, *FCE* foot core exercise

**Fig. 7:**
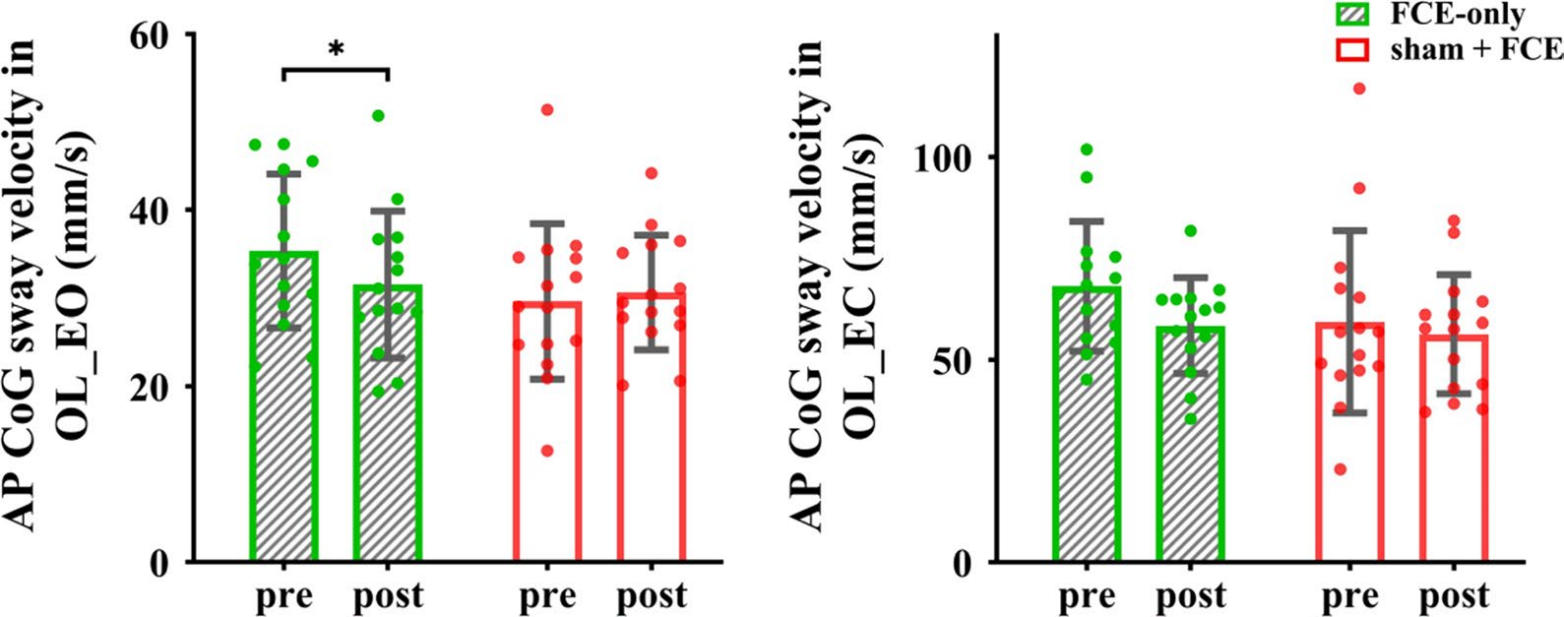
Comparisons of static balance between sham + FCE and FCE-only groups. *AP* anteroposterior, *CoG* center of gravity, *OL_EO* one-leg standing with eyes open, *OL_EC* one-leg standing with eyes closed, *FCE* foot core exercise; *p < 0.05

The performance in the sham + FCE and FCE-only groups was compared to explore the potential effects of sham on functional performance. For foot sensorimotor function, the results showed no significant interaction on toe flexor strength, MPJ flexor strength, and the passive ankle kinesthesia threshold (*p* = 0.320–0.923). Significant main effects of time were only observed for toe flexor strength (*F*_(1, 27)_ = 6.115, *p* = 0.020, ${\eta}_{\text{p}}^{2}$ = 0.185) and the passive kinesthesia threshold of PF (*F*_(1, 27)_ = 11.431, *p* = 0.002, ${\eta}_{\text{p}}^{2}$ = 0.297, Fig. 6). For static balance, a significant interaction was observed for the AP average CoG sway velocity in OL_EO (*F*_(1, 27)_ = 6.081, *p* = 0.020, ${\eta }_{p}^{2}$ = 0.184). However, post-hoc analysis showed that compared with the control group and baseline, the FCE-only group showed a significant decrease in the AP average CoG sway velocity in OL_EO (t = 2.715, *p* = 0.023, Cohen’s *d* = 0.69). In addition, a significant main effect of time was observed for the AP average CoG sway velocity in OL_EC (*F*_(1, 27)_ = 4.305, *p* = 0.048, ${\eta}_{\text{p}}^{2}$ = 0.138, Fig. 7).

## Discussion

This study aimed to investigate the effects of combining sensorimotor tDCS and FCE on the sensorimotor function of the foot (i.e., toe flexor strength and passive ankle kinesthesia) and static balance. Compared with the control group, tDCS + FCE could significantly improve the foot sensorimotor function. The findings suggested that implementing the intervention targeting the peripheral and central elements of the sensorimotor control is helpful, and it could ultimately benefit the functional performance.

The central nervous system plays a crucial role in regulating motor control and generating motor patterns, and positive changes in cerebral cortical neural activity could enhance physical performance [12]. tDCS has been shown to improve functional performance by modulating the neural excitability of the cerebral regions of the brain [27]. In recent studies, the intervention consisting of tDCS and other kinds of intervention (e.g., physical training) was used, and this kind of combined intervention yielded greater benefits on functional performances than using only one type of intervention (i.e., training or tDCS only) [17, 18, 28]. Bruce et al. observed that a 4-week tDCS combined with eccentric exercise facilitated tibialis anterior activation in people with CAI [17]. Besides, multi-session tDCS could enhance the effects of postural training on postural stability in older adults with high risk of fall [28]. The present study provided confirmatory but novel evidence that this type of intervention using tDCS in combination with FCE could augment the benefits on the sensorimotor function of the foot, even in healthy younger cohort.

One reason for the augmentation of the combined intervention is that this intervention could augment the functionalities in the peripheral and central elements of the sensorimotor control, thus inducing greater improvements than “single-target” intervention. Another potential reason behind the interaction between peripheral intervention (i.e., FCE) and central intervention (i.e., tDCS) may be that in addition to the improvements in peripheral muscular function, FCE could induce a reduction in short-interval intracortical inhibition (SICI), assisting in the production of voluntary force output by modulating descending excitatory drive (“bottom-up regulation”) [29, 30]. Furthermore, such reduction in SICI induced by FCE aligns with the same direction of the effects of tDCS (“top-down control”) on SICI (i.e., “top-down regulation”) [31]. Therefore, the “bottom-up control” by FCE and “top-down control” by tDCS align to reduce SICI, thus improving the foot sensorimotor function. Neuroimaging techniques (e.g., functional MRI) and neurophysiologic measurements (e.g., peripheral nerve conduction) are worthy to be used in future studies to explicitly characterize the peripheral and central elements of the sensorimotor pathway, and such knowledge could help further understand how this combined intervention works. Besides, previous studies showed that four weeks of tDCS combined with eccentric training could change neural plasticity by improving cortical excitability, which lasted over 6 weeks [17]. Even if the follow-up assessment was not collected in this study, however, it speculated that the improvements induced by this combined intervention may persist for a certain period. Future studies are needed to examine the long-term follow-up effects of this intervention.

No significant effect of the combined intervention was observed on static balance in this study. One potential reason is the stimulus target effect. Specifically, tDCS applied over the cerebellum, which is the main regulatory center of balance, may generate a more effective improvement in static balance than the sensorimotor cortex. Although a few studies reported that tDCS targeted sensorimotor showed positive effects on postural stability and balance, however, limited space for improvement may be available [28]. This reason may also explain the trend towards a significant association between the changes in MPJ flexor strength, an important contributor for balance control, and the AP average CoG sway velocity in OL_EC. The association suggested that in this healthy cohort, the improvements in the sensorimotor function of the foot may not necessarily turn into the improvement in the balance control performance, which was excellent before the intervention. Future studies are needed to explore the effects of this combined intervention on people with diminished or impaired functionality, such as those with foot injuries.

The potential effects of sham stimulation when using this combined type of intervention were also explored. Theoretically, sham stimulation only mimics the sensations on the scalp that is similar to tDCS, enabling successful blinding [32, 33]. However, an increasing number of studies demonstrated that the traditional inactive sham may potentially induce neurophysiologic changes in the brain. Boonstra et al. observed that the mean frequency of EEG was significantly reduced after receiving this kind of inactive sham, and it may potentially exert neuromodulatory effects [20]. Such effects may be increased in multiple-session interventions [32]. Therefore, in the present study, a reference group was included after the first two groups completed the intervention. The results showed that no placebo effects were observed, suggesting that this kind of sham stimulation may be a valid procedure to be implemented when designing studies by using similar types of combined intervention. Nevertheless, carefully examining the potential effects of sham in studies using tDCS is still necessary.

Several limitations should be noted. First, healthy young adults were recruited in this study. The effects of intervention combining HD-tDCS with FCE on foot sensorimotor function and static balance in adults with diminished or impaired functionality (e.g., stroke survivors, chronic ankle instability, and older adults with high fall risk) are worth exploring in future studies. Also, only a small sample of male adults was enrolled, future studies with a larger sample size of participants with similar numbers of men and women are needed. Second, only the immediate effects of the combined intervention were assessed. Future studies are needed to examine the long-term effects of this intervention. Third, only functional performance was measured. Exploring the effects of this intervention on the cortical activities within the brain is highly demanded in future studies. This direct neuroimaging evidence could help further understand the neurophysiologic pathways/mechanism underlying the effects of this intervention on the sensorimotor function of the foot and balance control.

## Conclusion

To the authors’ knowledge, this study was the first to examine the effects of intervention using tDCS in combination with foot core exercise to improve the sensorimotor function of the foot and static balance in healthy younger adults. The results showed that participating in 4 weeks of the combined intervention effectively enhanced toe flexor strength and foot–ankle sensory function compared with the control group, suggesting a promising novel strategy for the rehabilitation of foot function.

## Data Availability

All data generated or analysed during this study are included in this published article.

## Abbreviations

tDCS: Transcranial direct current stimulation
HD-tDCS: High-definition transcranial direct current stimulation
FCE: Foot core exercise
CAI: Chronic ankle instability
EEG: Electroencephalogram
PF: Plantarflexion
DF: Dorsiflexion
INV: Inversion
EV: Eversion
MPJ: Metatarsophalangeal joint
TL_EO: Two-leg standing with eyes open
TL_EC: Two-leg standing with eyes closed
OL_EO: One-leg standing with eyes open
OL_EC: One-leg standing with eyes closed
AP: Anteroposterior
ML: Medial–lateral
CoG: Center of gravity
SICI: Short-interval intracortical inhibition
MRI: Magnetic resonance imaging
